# Autoimmune Hepatitis and Systemic Lupus Erythematosus Overlap Syndrome: A Case Report and Literature Review

**DOI:** 10.7759/cureus.74962

**Published:** 2024-12-02

**Authors:** Maryam A Kheyami, Fatin R Almahroos, Alaa M Alzamrooni, Jawad Khamis, Ali Albaharna

**Affiliations:** 1 Gastroenterology and Hepatology, Salmaniya Medical Complex, Manama, BHR; 2 Internal Medicine, Salmaniya Medical Complex, Manama, BHR

**Keywords:** autoimmune hepatitis, deranged liver function tests, hepatic, immune-mediated disorders, jaundice, joint pain, liver histology, lupoid hepatitis, overlap syndrome, systemic lupus erythematosus

## Abstract

Autoimmune hepatitis-systemic lupus erythematosus (AIH-SLE) overlap syndrome is a rare disease entity. In some cases, a delay in the occurrence of overlap is observed, where the diagnosis of one of the conditions precedes the other. However, other patients have features of both disorders simultaneously upon initial presentation, leading to a direct diagnosis of AIH-SLE overlap syndrome. Thus, the highly variable and non-specific clinical presentations of AIH-SLE overlap syndrome pose a diagnostic challenge that can only be overcome by maintaining a high index of suspicion. The best clinical practice involves the consideration of a wide list of differential diagnoses for each case, tailored toward its unique combination of symptoms and clinical findings. Although challenging, the timely establishment of an accurate diagnosis is crucial as even the etiologies sharing similar autoimmune pathophysiology demonstrate varying responses to the different therapeutic agents. This case report summarizes seven years of a middle-aged female’s disease course, starting with her initial presentation, leading to her diagnosis with AIH, moving on to the establishment of overlap with SLE, and monitoring her response to the various treatment regimens she followed over the years. Despite trials of several recommended treatment regimens, the patient did not meet the requirements of remission for either AIH or SLE until the end of this observatory period. For the next steps in her management, several biological agents are under consideration as the existing scientific literature lacks recommendations specific to AIH-SLE overlap syndrome. This case report is intended to enrich the limited scientific literature pertaining to AIH-SLE overlap syndrome by documentation of this patient’s individual disease course and review of the existing literature.

## Introduction

Autoimmune hepatitis (AIH) is a rare disorder estimated to affect 15 out of every 100,000 of the global population, with a higher prevalence in females [1]. Similar to other causes of primary liver disease, AIH has a wide spectrum of clinical presentations. In around half of the cases, patients are asymptomatic with incidental findings of deranged liver function tests (LFTs) [2]. Other presentations include mild symptoms such as jaundice and pruritus or late-stage disease with liver cirrhosis and fulminant liver failure [3]. Moreover, several extra-hepatic manifestations have been reported in patients with AIH, ranging from non-specific manifestations such as transient arthralgia (around 35%) and cutaneous rash (8-17%), to limited autoimmune disorders such as thyroiditis (8-23%) and inflammatory bowel disease (2-8%), to systemic diseases such as rheumatoid arthritis (2-4%), SLE (1-2.6%), and mixed connective tissue disease (1-2.5%) [2]. Due to the non-specific presentation and biochemical features of AIH, a definitive diagnosis is often delayed until a histologic confirmation is obtained [2,3].

On the other hand, systemic lupus erythematosus (SLE) is an autoimmune multi-organ disease, with an estimated global prevalence of 44 individuals per 100,000 [4]. Similar to AIH, SLE can present with non-specific symptoms including fever, weight loss, and fatigue in addition to skin rashes and joint pain [5]. Furthermore, hepatic involvement is commonly observed through various mechanisms including lupus hepatitis, drug-induced hepatotoxicity, hepatic venous thrombosis, and concomitant primary sclerosing cholangitis [6]. In a study involving 238 patients with SLE, Runyon et al. documented the existence of liver disease in 21% [7].

An overlap between both disease entities, called AIH-SLE overlap syndrome, is considered rare [6]. It is estimated to affect 1-2.6% of patients with AIH and 1.3-4.7% of patients with SLE [2,6].

The distinction between AIH-SLE overlap syndrome, AIH and its other associated extra-hepatic manifestations, and hepatic disease in SLE is imperative considering the non-specific presentations and possible similarities in initial blood testing. This early distinction would allow the use of disease-specific treatments, reducing the risk of complications and achieving better overall patient outcomes.

## Case presentation

A 33-year-old Bahraini female was in her usual state of health until December 2016, when she presented to Salmaniya Medical Complex complaining of yellowish discoloration of her eyes for several days. This was associated with dark urine, nausea, and vomiting. She had no fever, abdominal pain, changes in bowel habits or stool color, history of blood transfusions, recent travel, alcohol consumption, or illicit drug use. She was married and in a monogamous relationship with a male partner. Her family history was unremarkable. 

On examination in the emergency department, she was hemodynamically stable, conscious, alert, oriented, and not in distress. Jaundice was evident in her sclera and facial skin. The examination was negative for clubbing, asterixis, bruising, or tattoos. Her abdomen was soft, lax, non-tender, with no hepatomegaly or splenomegaly. Other systems’ examination was unremarkable except for palpable left inguinal lymph nodes.

She was admitted and underwent laboratory investigations, which initially showed hyperbilirubinemia, deranged liver function tests (LFTs), raised inflammatory markers, and positive anti-nuclear antibodies. Further investigations showed negative viral screening and positive anti-Smith and anti-U1RNP antibodies, with low complement levels. The results are detailed in Table 1. An abdominal ultrasound was performed detecting no abnormalities. Thus, the patient was further investigated with a CT scan of the chest, abdomen, and pelvis, which noted diffuse lymphadenopathy mainly in the neck, both axillae, abdomen, and inguinal region (largest measuring 2 cm along the lesser curvature of the stomach, followed by left inguinal lymph node measuring 1.5 cm), with no evidence of hepatic abnormalities. Definitive diagnosis was not established in this admission, no treatment was initiated, and Rheumatology was not involved as the patient requested discharge against medical advice. Follow-up was arranged as an outpatient.

**Table 1:** Results of laboratory investigations during first presentation INR: international normalized ratio; ANA: anti-nuclear antibodies; anti-Sm: anti-Smith; anti-U1RNP: anti-U1 ribo-nucleo-protein; anti-JO1: anti-histydyl-tRNA synthetase; anti-RO/SSA: anti-Sjögren's syndrome-related antigen A; anti-LA/SSB: anti-Sjögren's syndrome-related antigen B; anti-SCL70: anti-topoisomerase I; anti-GPC: anti-gastric parietal cell; anti-LKM: anti-liver kidney mitochondrial; anti-SMA: anti-smooth muscle antibodies; C-ANCA: C-anti-neutrophil cytoplasmic antibodies; P-ANCA: perinuclear anti-neutrophil cytoplasmic antibodies; IgA: immunoglobulin A; IgG: immunoglobulin G; HIV: human immunodeficiency virus

Parameter	Value	Unit	Interpretation	Reference
White blood cell count	4.39	x10^9/L	Normal	3.6-9.6
Hemoglobin	11.2	g/dL	Low	12-14.5
Platelets	205	x10^9/L	Normal	150-400
INR	1.3		High	0.61-1.17
Total bilirubin	171	micromol/L	High	5-21
Direct bilirubin	120	micromol/L	High	0-5
Indirect bilirubin	51	micromol/L	High	≤18
Albumin	39	g/L	Normal	35-52
Alanine aminotransferase	1527	IU/L	High	≤33
Alkaline phosphatase	185	IU/L	High	50-136
Gamma-glutamyl transferase	463	IU/L	High	5-55
Erythrocyte sedimentation rate	60	mm/hr	High	≤20
C-reactive protein	15.1	mg/L	High	0-3
Rheumatoid factor	9.69	IU/m	Normal	≤14
ANA by immunofluorescence	Positive (ANA titer 1:640; speckled pattern)			
Anti-Sm antibodies	Positive			
Anti-U1RNP antibodies	Positive			
Anti-histone antibodies	Positive			
Anti-centromere antibodies	Negative			
Anti-JO1 antibodies	Negative			
Anti-RO/SSA antibodies	Negative			
Anti-LA/SSB antibodies	Negative			
Anti-SCL70 antibodies	Negative			
Anti-ribosomal antibodies	Negative			
Anti-GPC antibodies	Negative			
Anti-LKM antibodies	Negative			
Anti-SMA	Negative			
Anti-mitochondrial antibodies	Negative			
C-ANCA	Negative			
P-ANCA	Negative			
Myeloperoxidase antibodies	1.1	IU/mL	Negative	
Proteinase 3-ANCA	1.3	IU/mL	Negative	
Complement 3	20.7	mg/dL	Low	90-180
Complement 4	3	mg/dL	Low	10-40
Serum IgA	2.63	g/L	Normal	0.5-4
Serum IgG	35.7	g/L	High	5.4-16.5
Amylase	88	U/L	Normal	30-118
Hepatitis profile	Negative			
HIV antigens-antibodies	Negative			

**Figure 1:**
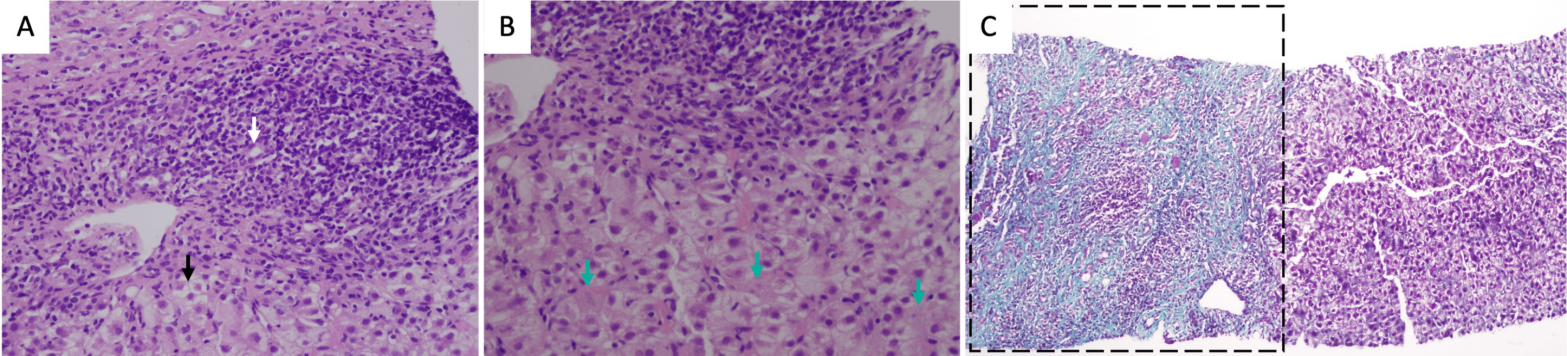
Microscopic view of the liver biopsy A: Moderate to marked portal inflammation including lymphoplasmacytic infiltrates (3/4); moderate to marked peri-portal interface hepatitis (3/4). Evident hepatocyte ballooning and rosette formation (black arrow), and ductular reaction (white arrow) (hematoxylin and eosin stain). B: Focal lytic necrosis (3/4) represented with (green arrows) (hematoxylin and eosin stain). C: Fibrous expansion of most portal areas with occasional portal-to-portal bridging (3/6) (Masson's trichrome stain). Other areas of the histological specimen showed focal confluent necrosis (1/6). No steatosis, duct injury, Mallory’s bodies, hemochromatosis (on the iron stain), alpha-1 antitrypsin bodies (on periodic acid–Schiff stain), dysplasia, or malignancy. Ishak scoring system [8]: Grade (total necro-inflammatory activity) = 10/18; Stage = 3/6 (portal fibrosis with occasional portal-to-portal bridging).

The patient was planned for an excisional inguinal lymph node biopsy during outpatient follow-up, which was inconclusive. Further histological investigation with a liver biopsy was prompted, which showed features compatible with autoimmune hepatitis (grade 10/18 and stage 3/6 on the Ishak scoring system), with no steatosis, duct injury, Mallory’s bodies, hemochromatosis (on iron stain), alpha-1 antitrypsin bodies (on periodic acid-Schiff stain), dysplasia, or malignancy (Figures 1A-1C) [8].

With a correlation of the patient’s presentation, laboratory investigations, and histological findings, a diagnosis of autoimmune hepatitis was established, and treatment with azathioprine 100 mg OD and prednisolone 5 mg OD was initiated.

In September 2017, she presented to the emergency department with a subjective fever for five days, associated with productive cough and vomiting. She denied a history of dyspnea, chest pain, abdominal pain, or changes in urinary or bowel habits. The physical examination was positive for bilateral basal crepitations and bilateral lower limb pitting edema extending to mid-shins. She underwent laboratory investigations, which revealed pancytopenia, direct hyperbilirubinemia, improvement of alanine aminotransferase (ALT), and worsening alkaline phosphatase (ALP) and gamma-glutamyl transferase (GGT) results compared to baseline and raised inflammatory markers. The results are detailed in Table 2. Her chest X-ray was unremarkable. She also underwent an abdominal ultrasound, which showed no biliary dilatation, no focal parenchymal mass lesion, with coarse parenchymal echotexture and heterogenous echogenicity of the liver (predominantly hypoechoic), consistent with autoimmune hepatitis. Therefore, she was admitted as AIH on azathioprine with a fever.

**Table 2:** Results of the laboratory investigations during the second presentation to the emergency department

On admission	Parameter	Value	Unit	Interpretation	Reference
	White blood cell count	3.15	x10^9/L	Low	3.6-9.6
	Hemoglobin	9.3	g/dL	Low	12-14.5
	Platelets	37	x10^9/L	Low	150-400
	Peripheral blood smear	Features of thrombocytopenia noted, schistocytes 0.4%			
	Total bilirubin	25	micromol/L	High	5-21
	Direct bilirubin	16	micromol/L	High	0-5
	Indirect bilirubin	9	micromol/L	Normal	≤18
	Albumin	29	g/L	Low	35-52
	Alanine aminotransferase	46	IU/L	High	≤33
	Alkaline phosphatase	491	IU/L	High	50-136
	Gamma glutamyl transferase	1009	IU/L	High	5-55
	Erythrocyte sedimentation rate	124	mm/hr	High	≤20
	C-reactive protein	42.6	mg/L	High	0-3
On discharge	White blood cell count	3.13	x10^9/L	Low	3.6-9.6
	Hemoglobin	8.1	g/dL	Low	12-14.5
	Platelets	41	x10^9/L	Low	150-400
	Total bilirubin	10	micromol/L	Normal	5-21
	Albumin	25	g/L	Low	35-52
	Alanine aminotransferase	26	IU/L	Normal	≤33
	Alkaline phosphatase	270	IU/L	High	50-136
	Gamma glutamyl transferase	433	IU/L	High	5-55
	C-reactive protein	35.6	mg/L	High	0-3

Considering the immune-suppressed state of the patient, the treatment plan included starting empirical antibiotics and the discontinuation of azathioprine. Her partial septic workup was negative, so the dose of prednisolone was increased to 10 mg OD during admission, and ursodeoxycholic acid 250 mg TDS was started.

After 10 days of treatment, the patient’s LFTs showed gradual improvement with no improvement in terms of her pancytopenia (as detailed in Table 2), and she continued having spikes of fever. However, the patient insisted on leaving against medical advice and was discharged on prednisolone 5 mg OD and oral antibiotics.

The patient then traveled to a hospital abroad for a second opinion. She also reported a history of polyarthralgia associated with early morning joint stiffness for three months and weight loss of around 10 kg since her initial symptoms started. She denied any history of SOB, dysphagia, oral ulcers, hematuria, Reynaud’s phenomenon, thromboembolic events, history of abortion, or psychotic symptoms. Thus, a suspicion of connective tissue disease besides AIH prompted further investigations.

A diagnosis of SLE was then established based on the patient’s history and positivity of anti-nuclear antibodies, anti-double stranded DNA, anti-beta-2 glycoprotein IgM, and anti-cardiolipin IgM, in addition to low complement levels. Therefore, she received pulse steroids for three days, followed by a steroid tapering dose. Hydroxychloroquine was initiated, and her previous regimen of azathioprine was restarted. One day later, she presented with acute hearing loss, giddiness, and left ear pain. Audiogram testing was positive for left-sided sensorineural hearing loss, which was attributed to hydroxychloroquine - a rare side effect [9].

Over the two years following her diagnosis with AIH-SLE overlap syndrome, trials of achieving adequate maintenance included treatment courses combining prednisolone and hydroxychloroquine with either azathioprine or mycophenolate mofetil. This was followed by two years of clinical and biochemical remission on a hydroxychloroquine-only treatment regimen. Negativity of SLE-related autoantibodies was also achieved.

The patient maintained remission until 2022 when she developed another flare, marked with clinically evident jaundice and derangement of LFTs (ALP 191 U/L, ALT 1049 U/L, AST 1222 U/L, GGT 740 U/L, total bilirubin 88 micromol/L) and elevation of alpha-fetoprotein (71.4 micrograms/L). Accordingly, a triphasic abdominal CT was done and revealed subtle wall surface irregularity along with stigmata of portal hypertension, with no cirrhosis or focal lesions. Malignancies, steatohepatitis, and cholelithiasis were ruled out by imaging, and viral hepatitis was ruled out by serology. She was started again on prednisolone (initially 60 mg OD with tapering) and azathioprine 150 mg OD. Hydroxychloroquine 200 mg BD was continued.

A few months later, azathioprine was stopped in view of leukopenia and the patient developed another flare. Subsequently, she was kept on triple therapy of mycophenolate mofetil 1 gram BD, hydroxychloroquine 200 mg BD, and prednisolone (minimum dose of 10 mg OD as further tapering resulted in further elevation of LFTs). However, in view of the insufficient response to previous treatment regimens, the patient is currently being planned for escalation to biological immunotherapy by rituximab.

**Figure 2:**
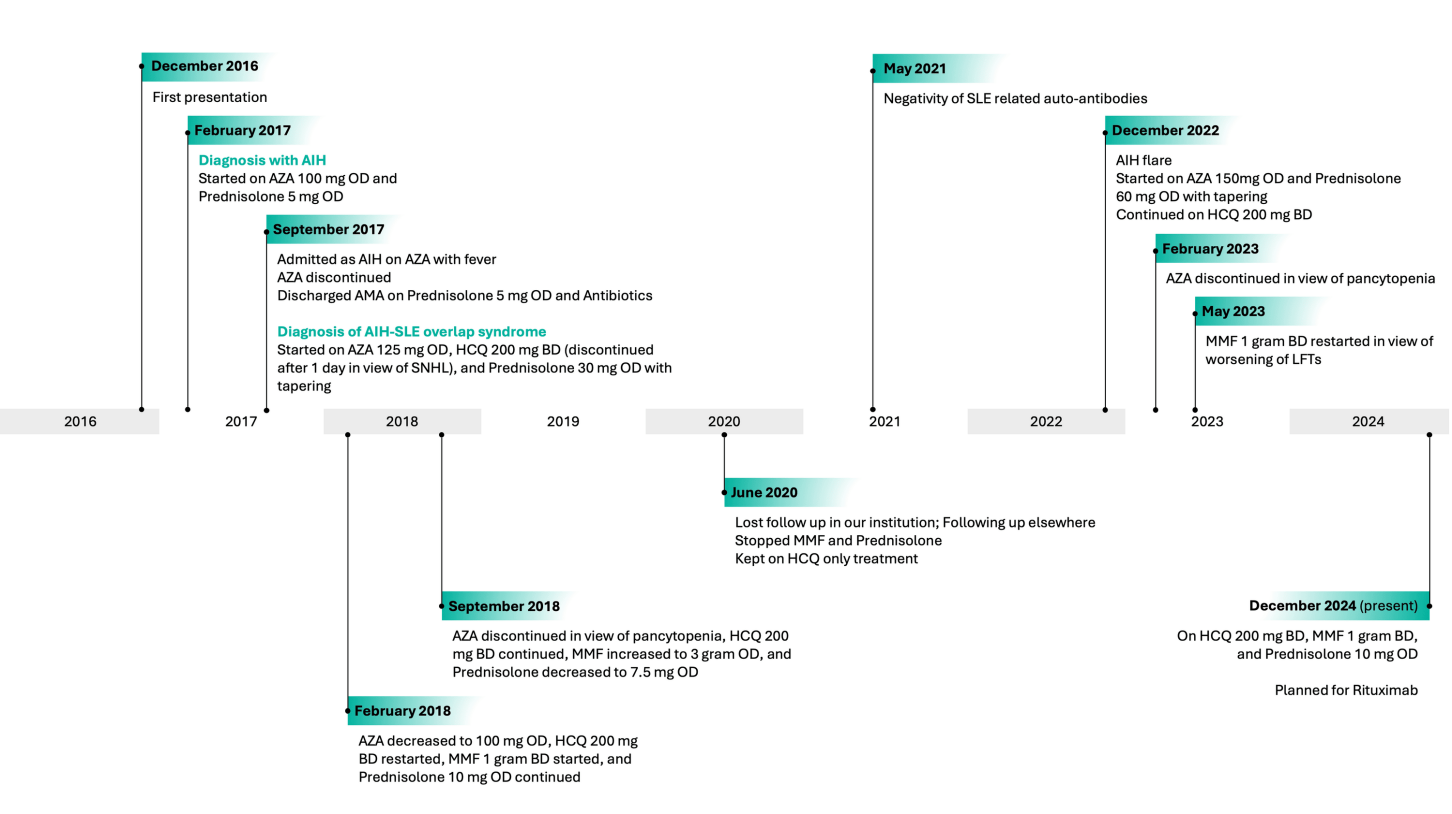
Outline of the patient's disease course and treatment regimens AIH: autoimmune hepatitis; AZA: azathioprine; AMA: against medical advice; SLE: systemic lupus erythematosus; HCQ: hydroxychloroquine; SNHL: sensori-neural hearing loss; MMF: mycophenolate mofetil

The complex course of this patient’s disease and the different treatment regimens prescribed during eight years of follow-up are outlined in Figure 2.

Literature review

Autoimmune Hepatitis

Autoimmune hepatitis can be presumed as a diagnosis based on the clinical suspicion and positivity of typical serological markers, although histological assessment by liver biopsy is often required to confirm the diagnosis [3]. This prompted the development of several diagnostic guidelines to assess the probability of AIH in individual patients and standardize its diagnosis for research purposes. The International Autoimmune Hepatitis Group proposed simplified criteria for AIH diagnosis, incorporating laboratory investigations, auto-antibodies, and histological findings on liver biopsy (Table 3) [10]. 

**Table 3:** Simplified diagnostic criteria of the International Autoimmune Hepatitis Group Simplified diagnostic criteria of the International Autoimmune Hepatitis Group [10]: Score of ≥7 = definite AIH; score of ≥6 = probable AIH *Addition of points achieved for all autoantibodies (maximum, two points). a: Atypical features on liver histology = signs of another liver disease e.g., steatohepatitis. b: Compatible features on liver histology = chronic hepatitis with lymphocytic infiltration without all the features considered typical. c: Typical features on liver histology = presence of all of the following, portal lymphocytic/lymphoplasmacytic mononuclear cell infiltration extending into the lobule, interface hepatitis, emperipolesis, and hepatic rosette formation. ANA: anti-nuclear antibodies; anti-SMA: anti-smooth muscle antibodies; anti-LKM: anti-liver kidney mitochondrial; anti-SLA/LP: anti-soluble liver antigen/liver pancreas

Feature/parameter	Discriminator	Score
ANA or anti-SMA +	≥1:40	+1*
	≥1:80	+2*
Or anti-LKM antibodies +	≥1:40	+2*
Or anti-SLA/LP antibodies +	Any titer	+2*
IgG or 𝛾-globulin level	>upper limit of normal	+1
	>1.1 x upper limit of normal	+2
Liver histology (evidence of hepatitis is a necessary condition)	Atypical ^a^	0
	Compatible with AIH ^b^	+1
	Typical of AIH ^c^	+2
Absence of viral hepatitis	No	0
	Yes	+2

In clinical practice, AIH can be further divided into three sub-types, with distinct serological findings, i.e., type 1, type 2, and autoantibody-negative autoimmune hepatitis. The most common subtype is type 1 AIH, accounting for around two-thirds of the disease burden and affecting both pediatric and adult populations [2,3]. It is characterized by the positivity of anti-nuclear antibodies (ANA), anti-smooth muscle antibodies (ASMA), anti-actin antibodies (AAA), and atypical perinuclear anti-neutrophil cytoplasmic antibodies (P-ANCA) [3]. On the other hand, type 2, AIH which predominantly affects children, is characterized by liver-kidney microsomal-1 antibodies (LKM-1), anti-soluble liver antigen/liver pancreas antibodies (anti-SLA/LP), and anti-liver cytosol-1 antibodies (ALC-1) [2,3]. Out of the antibodies associated with type 2 AIH, LKM-1 has been linked to worse prognostic outcomes [2]. In around 20% of patients with AIH, the negativity of the serological markers typical for AIH is observed, classifying them as autoantibody-negative autoimmune hepatitis [3]. In this group, AIH is suggested by the patients’ clinical and histopathological features and response to anti-inflammatory therapy [3]. 

Regardless of the subtype, the recommended initial treatment of AIH is high-dose glucocorticoid-only therapy or glucocorticoid plus antimetabolite (such as azathioprine or mycophenolate mofetil) for patients at increased risk of high-dose glucocorticoid side effects [11]. In recent studies, glucocorticoid plus mycophenolate mofetil provided better outcomes including higher rates of response to treatment and lower rates of side effects than glucocorticoid plus azathioprine therapy [11]. Adjustment of doses or treatment regimen is considered in non-responders (patients with <50% reduction of aminotransferase levels from baseline within four weeks of treatment initiation) [11]. Moreover, initial non-responders who achieve biochemical remission with subsequent adjustments to treatment will eventually require long-term maintenance therapy [11]. According to the International Autoimmune Hepatitis Group, remission is defined as a complete biochemical response (normalization of serum aminotransferase and IgG levels) within six months from treatment initiation and histologic remission (hepatitis activity index <4 on liver biopsy) [11]. However, repeating liver biopsy in a patient who achieved a complete biochemical response is not necessary to confirm remission and is rarely done in clinical settings [11]. Patients who remain non-responding even after maximization of medical therapy have a higher risk of developing AIH complications, including portal hypertension, liver cirrhosis, and hepatocellular carcinoma, and thus are candidates for liver transplantation [11]. Nevertheless, studies have estimated the recurrence of AIH post-transplantation to be 20% after five years and 31% after 10 years [11]. 

Systemic Lupus Erythematosus

SLE is a systemic connective tissue disorder, with a wide range of non-specific presentations related to the disease process. Thus, a high index of suspicion of SLE based on clinical features prompts further serologic investigations to confirm the diagnosis, as recommended by the 2019 EULAR/ACR classification criteria - summarized in Table 4 [5]. According to the mentioned criteria, ANA positivity and at least one clinical criterion are prerequisites for any SLE diagnosis [5]. 

**Table 4:** European Alliance of Associations for Rheumatology (EULAR)/ American College of Rheumatology (ACR) classification criteria for Systemic Lupus Erythematosus 2019 EULAR/ACR classification criteria for SLE [5]: total score ≥10 with one or more clinical criterion (and ANA positivity) = diagnosis of SLE. HEp-2: human epithelial protein type-2 *Additional additive (clinical or immunology) criteria are counted towards the total score as follows: Do not count a criterion if there is a more likely explanation than SLE; occurrence of a criterion on at least one occasion is sufficient; criteria need not occur simultaneously; within each domain (e.g., mucocutaneous, complement proteins), only the highest-weighted criterion is counted toward the total score if more than one is present.

Entry criterion (necessary to classify SLE)		
ANA (titer ≥1:80 on HEp-2 cells or an equivalent positive test)		
Clinical domain and criteria (at least one clinical criterion required to classify SLE)*		
Clinical domains	Clinical criteria	Weight
Constitutional	Fever	2
Hematologic	Leukopenia	3
	Thrombocytopenia	4
	Autoimmune hemolysis	4
Neuropsychiatric	Delirium	2
	Psychosis	3
	Seizure	5
Mucocutaneous	Nonscarring alopecia	2
	Oral ulcers	2
	Subacute cutaneous or discoid lupus	4
	Acute cutaneous lupus	6
Serosal	Pleural or pericardial effusion	5
	Acute pericarditis	6
Musculoskeletal	Joint involvement	6
Renal	Proteinuria >0.5 g per 24 hours	4
	Renal biopsy class II or V lupus nephritis	8
	Renal biopsy class III or IV lupus nephritis	10
Immunology domains and criteria		
Immunology domain	Immunology criteria	Weight
Antiphospholipid antibodies	Anti-cardiolipin antibodies OR lupus anticoagulant OR anti-beta2-glycoprotein	2
Complement proteins	Low C3 OR low C4	3
	Low C3 AND low C4	4
SLE-specific antibodies	Anti-dsDNA antibodies OR anti-Smith antibodies	6

The basis of pharmacological treatment in SLE is hydroxychloroquine for all patients [12]. However, the recommended approach to SLE management involves a personalized assessment that takes into consideration the disease activity, organ involvement, patients’ comorbidities, response to previous treatments (if any), and patients’ preferences [12]. Based on these factors, additional treatment with non-steroidal anti-inflammatory drugs, glucocorticoids, other immune modulators (e.g., azathioprine, methotrexate, and mycophenolate mofetil), and/or biologic treatments (e.g., belimumab and anifrolumab) is considered [12]. The goal of therapy in SLE is to achieve remission or low disease activity, to improve the patients’ quality of life, and to prevent organ damage while minimizing therapeutic side effects [12]. There is no consensus regarding the definition of SLE remission and low disease activity. However, the Definitions of Remission in SLE (DORIS) Task Force defined remission as a score of 0 on the Systemic Lupus Erythematosus Disease Activity Index (SLEDAI) and <0.5 on physician global assessment (PGA) [12]. According to the DORIS Task Force, remission includes patients on low-dose glucocorticoids (e.g., prednisolone ≤5 mg/day), maintenance doses of hydroxychloroquine, and/or maintenance doses of immune suppressants [12]. Similarly, the Lupus Low Disease Activity State (LLDAS) proposes SLEDAI-2K score of ≤4 (with no major organ system activity and no new-onset symptoms compared to previous assessments), a PGA score of ≤1, and negative serology [12]. Patients can be on prednisolone ≤7.5 mg per day, maintenance doses of hydroxychloroquine, and/or maintenance doses of immunosuppressive therapy [12].

The extent of systemic involvement and severity of SLE activity are the major contributors to morbidity in SLE. However, patients with SLE are at increased risk of malignancies, especially non-Hodgkin’s lymphoma (three-fourfold increased risk compared to the general population) [12]. Moreover, patients with SLE have a higher incidence of osteoporosis, osteonecrosis, and recurrent infections, which is partially attributed to the pharmacological treatments required to control the disease [12]. 

Differential Diagnoses

The isolated finding of deranged LFTs on laboratory testing is a non-specific marker of hepatic disorders, encompassing acute disorders (such as acute viral hepatitis, acute drug-induced liver injury, hepatic ischemia, etc.) and chronic disorders (such as autoimmune hepatitis, non-alcoholic fatty liver disease, primary sclerosing cholangitis, etc.) [3]. Thus, further investigations along with a proper detailed history are almost always required to narrow down the differential diagnoses.

In the case presented in this report, the patient later developed concurrent constitutional symptoms and joint pain, prompting the consideration of a broader list of differentials. Possible differential diagnoses of this patient considering her presentation and pre-established diagnosis of AIH include non-specific joint pain resembling extra-hepatic manifestations of AIH, AIH-SLE overlap syndrome, and AIH with other concurrent connective tissue disease. The prevalence of these disorders in patients diagnosed with AIH is estimated to be around 35%, 1-2.6%, and 1-7%, respectively [2]. The differential diagnoses considered in this patient and other differential diagnoses of joint pain and deranged LFTs are compared in Table 5.

**Table 5:** Comparison between possible differential diagnoses AIH: autoimmune hepatitis; ANA: anti-nuclear antibodies; anti-SMA: anti-smooth muscle antibodies; anti-SLA/LP: anti-soluble liver antigen/liver-pancreas; anti-LKM-1: anti-liver kidney mitochondrial-1; SLE: systemic lupus erythrematousus; anti-Sm: anti-Smith; RA: rheumatoid arthritis; RF: rheumatoid factor; anti-CCP: anti-cyclic citrullinated peptide; NSAIDs: non-steroidal anti-inflammatory drugs; NAFLD: non-alcoholic fatty liver disease; PSC: primary sclerosing cholangitis; P-ANCA: perinuclear anti-neutrophil cytoplasmic antibodies

Diagnosis	Characteristic clinical features	Liver function tests	Viral hepatitis profile	Serological markers	Histological findings
AIH with non-specific joint pain	Transient; Mostly coinciding with AIH flares [2]	↑	Negative	Antibodies associated with type 1 AIH (e.g., ANA, anti-SMA) [3]; Antibodies specific for type 2 AIH (e.g., anti-SLA/LP, anti-LKM-1 antibodies)[3]	Typical features of autoimmune hepatitis including portal mononuclear cell infiltration(predominantly lymphoplasmacytic), interface hepatitis, emperipolesis, hepatic rosette formation [10,13]
AIH-SLE overlap syndrome	-	↑	Negative	Antibodies associated with type 1 AIH (e.g., ANA, anti-SMA) [3]; Antibodies specific for type 2 AIH (e.g., anti-SLA/LP, anti-LKM-1 antibodies) [3]; SLE-specific antibodies (anti-double stranded DNA antibodies, anti-Sm antibodies) [5]	Features of autoimmune hepatitis
AIH and other connective tissue disease (e.g., RA)	Joint swelling [2]; Other characteristic physical findings of underlying connective tissue disease (e.g., typical findings of RA)	↑	Negative	Antibodies associated with type 1 AIH (e.g., ANA, anti-SMA) [3]; Antibodies specific for type 2 AIH (e.g., anti-SLA/LP, anti-LKM-1 antibodies) [3]; Connective-tissue disease-specific antibodies	Features of autoimmune hepatitis
Lupus hepatitis	Mucocutaneous manifestations of SLE (most commonly malar rash) [5]	↑	Negative	Anti-ribosomal P antibodies [6]; SLE-specific antibodies (anti-double stranded DNA antibodies, anti-Sm antibodies) [5]	Periportal lymphocytic infiltration, with isolated areas of necrosis [6]
SLE with drug-induced hepatotoxicity	Acute drugs toxicity; History of hepatotoxic drug use in the treatment of SLE (e.g., NSAIDs, azathioprine, mycophenolate mofetil, cyclophosphamide, and rarely hydroxychloroquine)[14]; Mucocutaneous manifestations of SLE (most commonly malar rash) [5]	↑; ALT >1,000 IU/L (in acute insults) [15]	Negative	SLE-specific antibodies (anti-double stranded DNA antibodies, anti-Sm antibodies) [5]	Non-specific features of hepatic injury correlating to the causative agent [13]
SLE with viral hepatitis	History of recent travel, unprotected sexual contact, IV drug abuse, tattoo, and piercing [16]; Mucocutaneous manifestations of SLE (most commonly malar rash) [5]	↑; ALT >1,000 IU/L (in acute phase) [15]	Positive	Cryoglobulinemia (mostly associated with hepatitis C) [17]; SLE-specific antibodies (anti-double stranded DNA antibodies, anti-Sm antibodies) [5]	Leukocyte infiltration (predominantly lymphocytic), hepatocellular damage, Kupffer-cell activation, and bile duct distortion [13]
SLE with NAFLD	Increased probability with metabolic diseases (e.g., diabetes mellitus, dyslipidemia) [18]; Mucocutaneous manifestations of SLE (most commonly malar rash) [5]	↑	Negative	SLE-specific antibodies (anti-double stranded DNA antibodies, anti-Sm antibodies) [5]	Macrovesicular and microvesicular steatosis, lipogranulomas, megamitochondria, ballooning of hepatocytes, Mallory‐Denk bodies, features of chronic portal inflammation [13]
SLE with alcoholic fatty liver disease	History of alcohol abuse; Mucocutaneous manifestations of SLE (most commonly malar rash) [5]	↑; AST:ALT ratio more than two [19]	Negative	SLE-specific antibodies (anti-double stranded DNA antibodies, anti-Sm antibodies) [5]	Features similar to NAFLD, in addition to polymorphonuclear cells surrounding Mallory-Denk bodies and sclerosing hyaline necrosis [13]
SLE with hepatic venous thrombosis	History of previous thrombotic events (e.g., recurrent abortions in females, DVT, PE), or established diagnosis of concomitant antiphospholipid syndrome [5,20]; Mucocutaneous manifestations of SLE (most commonly malar rash) [5]	↑	Negative	Antiphospholid syndrome related antibodies (anti-cardiolipin antibodies, lupus anticoagulant, anti-beta2-glycoprotein) [5]; SLE-specific antibodies (anti-double stranded DNA antibodies, anti-Sm antibodies) [5]	Sinusoidal dilation, portal tract fibrosis, bile-ductular reaction, centrilobular hepatocyte necrosis, and features of chronic inflammation [13]
SLE with PSC	Mucocutaneous manifestations of SLE (most commonly malar rash) [5]	↑; predominantly cholestatic pattern [21]	Negative	P-ANCA antibodies [21]	Periductal edema and concentric fibrosis(often described as onion skin fibrosis), ductal proliferation, small duct atrophy, and features of chronic inflammation [13]

## Discussion

Considering the initial presentation of this patient with jaundice and no other systemic symptoms, various causes of hepatic dysfunction were suspected and investigated accordingly. As the laboratory investigations showed deranged LFTs, positivity of anti-nuclear antibodies, and negativity of viral hepatitis screen, further investigation by liver biopsy was sought. By applying these findings to the simplified diagnostic criteria of the International Autoimmune Hepatitis Group, the patient had a score of seven (ANA ≥1:80 = two points, IgG > 1.1x upper limit of normal = two points, compatible liver histology = one point, and absence of viral hepatitis = two points), which constitutes definite type 1 AIH [10].

It is worth noting that at the stage of diagnosis with AIH, the patient had a positive ANA, anti-Sm, anti-U1RNP, and anti-histone antibodies, which are commonly associated with connective tissue diseases including SLE [5,22]. However, differential diagnoses of systemic diseases were missed at that point as the patient’s presentation with jaundice and deranged LFTs was explained by her hepatic disorder.

Similarly, in the patient’s second presentation with fever and pancytopenia, a peripheral blood smear confirmed thrombocytopenia and an insignificant percentage of schistocytosis. These findings are also documented in SLE and constitute a part of the 2019 EULAR/ACR classification criteria [5]. However, a more probable explanation for these findings was azathioprine-induced pancytopenia and resultant infection. For such cases, the 2019 EULAR/ACR classification criteria state that a clinical or immunological criterion should not be scored if another differential diagnosis is more probable than SLE [5]. Hence, with no other clinical or immunological features to suggest SLE, this patient did not fulfill EULAR/ACR criteria for SLE [5]. 

A significant development in the disease course was when the patient started complaining of joint pain with chronic weight loss. This raised the clinical suspicion of SLE and other connective tissue disorders. The new-onset clinical features and positivity of SLE-specific antibodies constitute a score of 24 on the 2019 EULAR/ACR classification criteria (fever = two points, thrombocytopenia = four points, joint involvement = six points, antiphospholipid antibodies = two points, low C3 and C4 = four points, SLE-specific antibodies = six points), establishing a diagnosis of SLE [5]. Moreover, the patient’s autoantibody profile rules out other connective tissue disorders and non-specific joint pain as extrahepatic manifestations of AIH. The fulfillment of the criteria of diagnosis of both AIH and SLE concomitantly in this patient established her diagnosis with type 1 AIH and SLE overlap syndrome. 

Among the subtypes of AIH, type 1 (as in this case) has a more documented incidence with AIH-SLE overlap syndrome in the scientific literature [23-25]. However, the relative prevalence of each subtype in AIH-SLE overlap syndrome cannot be estimated due to the limited published data. Moreover, although AIH is often classified into subtypes in the literature, these have not been established as distinct clinical or pathological entities, questioning the significance of such classification in clinical settings [3]. 

The treatment regimen followed in this patient underwent several changes based on the patient’s response and tolerance to treatment. Upon the initial diagnosis of isolated AIH, azathioprine and prednisolone were started, which was the recommended first-line management at the time, although more recent data proved better efficacy of mycophenolate mofetil [11]. A few months later, azathioprine therapy was paused due to the development of pancytopenia and then restarted when AIH-SLE overlap syndrome was established along with initiating hydroxychloroquine and glucocorticoids. The initial treatment regimen of SLE (hydroxychloroquine and prednisolone) is also in line with the recommendations set for SLE management [12]. During follow-up, a remarkable finding in this patient is the maintenance of remission for two years on a hydroxychloroquine-only regimen, which is not incorporated within AIH guidelines. This supports the limited studies suggesting the efficacy of hydroxychloroquine in maintaining remission in AIH [26]. However, subsequent flares of AIH prompted the escalation to high-dose glucocorticoids and hydroxychloroquine, in addition to either azathioprine or mycophenolate mofetil.

Despite these modifications, the patient was unable to achieve complete normalization of LFTs, which is the hallmark of remission in AIH [11]. Moreover, since prednisolone could not be tapered below 10 mg per day without worsening the patient’s LFTs, she cannot be assessed for SLE remission or low disease activity as outlined by DORIS and LLDAS, respectively [12]. Therefore, escalation of treatment to include biological therapies is under consideration for this patient. Several clinical trials addressed the use of biologics in the treatment of AIH and SLE individually. Out of these agents, rituximab (which is considered in this patient) and belimumab showed positive results in the management of both AIH and SLE in separate studies [12,27-30]. However, there were no scientific studies recommending these agents (or other biologics) in the context of AIH-SLE overlap syndrome in our literature review.

## Conclusions

This case report outlines the complex series of a patient’s clinical presentations during which the diagnosis developed from AIH to the co-existence of SLE, comprising AIH-SLE overlap syndrome. It highlights the importance of vigilance and a high index of suspicion towards new-onset clinical features arising after a diagnosis has been confirmed. In turn, this allows earlier diagnosis and disease-specific management, which would improve disease prognosis and overall patient outcomes.

In addition, the long follow-up period demonstrated in this report provides insight into the dynamic nature of chronic disease management in clinical settings where the interplay of patients’ tolerance, preference, and response to medications determines the management approach.

A limitation observed during the formulation of this case report is the scarcity of scientific literature and the absence of clinical guidelines specific to AIH-SLE overlap syndrome. Therefore, the mainstay of management of AIH-SLE overlap syndrome at present is the combination of recommendations for both AIH and SLE. However, the treatment regimen providing the most efficacy in the treatment of AIH-SLE overlap syndrome is yet to be determined.
